# Transudative chylothorax associated with alcoholic cirrhosis

**DOI:** 10.1093/omcr/omz019

**Published:** 2019-03-29

**Authors:** Yusuke Koshima, Shinichi Miyazaki, Jun Ninomiya, Yasumasa Kuno, Takuya Ikeda

**Affiliations:** 1 Junior Resident, Yokkaichi Municipal Hospital, Sibata2-2-37, Shibata, Yokkaichi-shi, Mie, Japan; 2 Department of Respiratory Medicine, Yokkaichi Municipal Hospital, Sibata2-2-37, Shibata, Yokkaichi-shi, Mie, Japan; 3 Department of Gastroenterology, Yokkaichi Municipal Hospital, Sibata2-2-37, Shibata, Yokkaichi-shi, Mie, Japan

## Abstract

We present the case of a 53-year-old man with decompensated alcoholic cirrhosis who was referred for right pleural effusion. After investigation, the patient was ultimately diagnosed with cirrhotic chylothorax. Chylothorax is a rare manifestation of cirrhosis, which results from the trans-diaphragmatic passage of chylous ascites. While chylothorax generally results in an exudative pleural effusion, cirrhotic chylothorax is always a transudative effusion. Biochemical characteristics are useful for diagnosis, avoiding potentially harmful diagnostic and therapeutic procedures.

Chylothorax, defined as a pleural fluid triglyceride (TG) level >110 mg/dl in the correct clinical context, is the accumulation of chyle in the pleural space. Malignancy and surgical trauma are the leading causes of chylothorax [1]. Because pleural fluid analysis is usually an exudative effusion with a predominance of lymphocytes, transudative chylothorax is a rare clinical entity.

A 53-year-old man with alcoholic cirrhosis was referred to us because of right pleural effusion. Eight months prior to our evaluation, the patient had previously presented with bilateral pleural effusion, ascites and lower extremity edema and, after extensive investigation, he was diagnosed with decompensated alcoholic cirrhosis. The patient had responded well to diuretic agents with resolution of the dyspnea and abdominal distention. However, a few months prior, the patient became diuretic-resistant, and developed exacerbation of the symptoms.

The patient was afebrile, with a heart rate of 100 beats/min, BP 97/62 mm Hg, and oxygen saturation of 94% on room air. Clinical examination revealed reduced air entry into the lower right hemithorax, abdominal distension, shifting dullness, and leg edema. A complete blood count was normal except for a hemoglobin concentration of 12.2 g/dl (normal range 13.7–16.8 g/dl). The results of a metabolic panel were normal except for serum sodium level of 129 mEq/l (normal range 138–145 mEq/l), a cholinesterase level of 91 IU/l (normal range 240–486 IU/l), and an albumin level of 2.2 g/dl (normal range 4.1–5.1 g/dl). The international normalized ratio was 1.3. Chest CT imaging demonstrated right predominant, but bilateral pleural effusion without significant parenchymal abnormalities and abdominal and pelvic CT was significant for a shrunken nodular liver, massive ascites and splenomegaly (Fig. 1a). Thoracocentesis yielded turbid pleural fluid (Fig. 1b above) with a protein level of 1.9 g/dl (serum protein, 6.2 g/dl, normal range 6.5–8.1 g/dl), lactate dehydrogenase (LDH) of 71 IU/l (serum LDH, 151 mg/dl, normal range 124–224) and TG level of 207 mg/dl (serum TG, 95 mg/dl, normal range 30–149 mg/dl); all of which were suggestive of chylothorax. Cultures and cytology were negative. Subsequent paracenteses revealed a milky fluid (Fig. 1b below) with a white-cell count of 736/μl (with 88% lymphocyte), albumin of 1.6 g/dl (serum albumin, 2.8 g/dl), protein of 3.3 g/dl, LDH of 101 IU/l and triglyceride level of 792 mg/dl that was also cytology and culture negative. The patient was ultimately diagnosed with cirrhotic chylothorax. Cell-free and concentrated ascites reinfusion therapy (CART) was administered to relieve symptoms caused by massive ascites, with eventual resolution of the pleural effusion.

**Figure 1:**
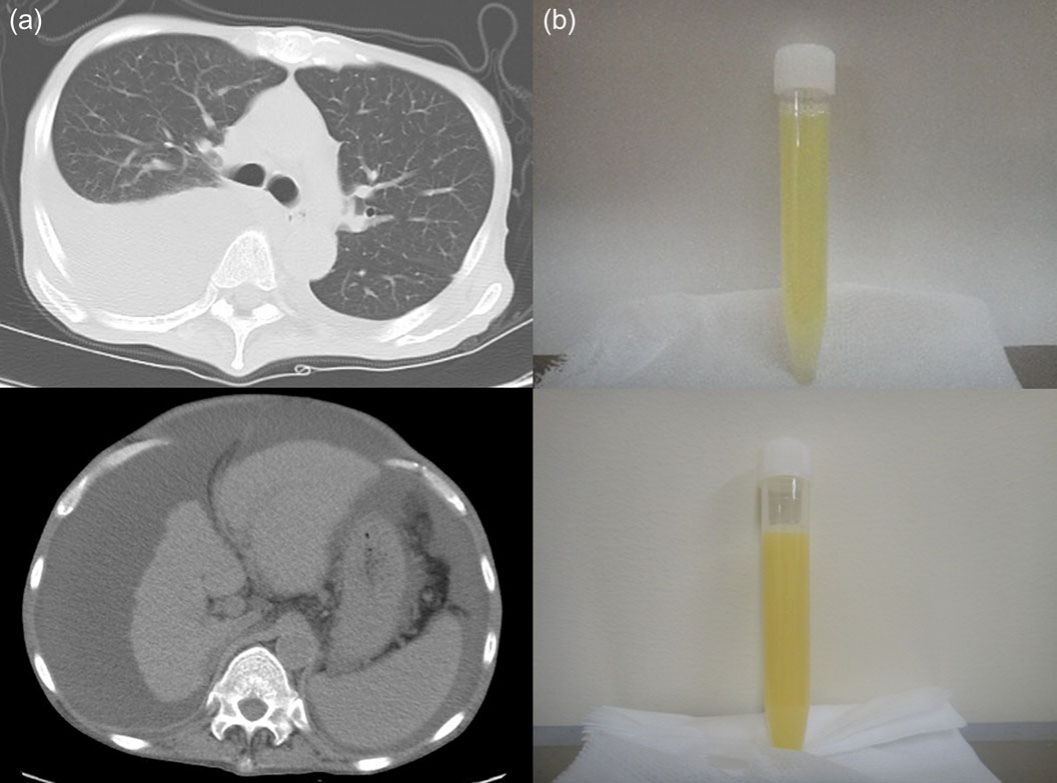
(**a**) CT imaging of the thorax and abdomen; (**b**) appearance of right pleural effusion (upper) and ascites (below).

Chylothorax is a rare manifestation of cirrhosis, which results from the trans-diaphragmatic passage of chylous ascites. While chylothorax generally results in an exudative pleural effusion, cirrhotic chylothorax is always a transudative effusion [2]. Transudative chylothorax has been reported in a small proportion of patients with amyloidosis, nephrotic syndrome, and superior vena cava obstruction, etc [3]. Therefore, biochemical characteristics are useful for diagnosis, avoiding potentially harmful diagnostic and therapeutic procedures. The management of chylothorax generally involves pleural drainage, dietary modification (oral or enteral low-fat diet, and total parenteral nutrition), treatment of the underlying disease, somatostatin analogs, and definitive intervention (pleurodesis, thoracic duct embolization or disruption, and thoracic duct ligation). In cirrhotic chylothorax, embolization, disruption, or ligation of the thoracic duct almost invariably worsens chylous ascites. Transjugular intrahepatic portosystemic shunt may be effective in refractory chylothorax due to liver cirrhosis [4].
